# Genome-wide association study (GWAS) for morphological and yield-related traits in an oil palm hybrid (*Elaeis oleifera x Elaeis guineensis*) population

**DOI:** 10.1186/s12870-019-2153-8

**Published:** 2019-12-03

**Authors:** Jaime A. Osorio-Guarín, Gina A. Garzón-Martínez, Paola Delgadillo-Duran, Silvio Bastidas, Leidy P. Moreno, Felix E. Enciso-Rodríguez, Omar E. Cornejo, Luz Stella Barrero

**Affiliations:** 1 Tibaitatá Research Center, Corporación Colombiana de Investigación Agropecuaria, Agrosavia, Bogotá, Colombia; 2 Palmira Research Center, Corporación Colombiana de Investigación Agropecuaria, Agrosavia, Palmira, Colombia; 3 School of Biological Sciences, Washington State University, Pullman, WA USA

**Keywords:** Association mapping, *Elaeis guineensis*, *Elaeis oleifera*, Genotyping-by-sequencing, Plant architecture, Yield

## Abstract

**Background:**

The genus *Elaeis* has two species of economic importance for the oil palm agroindustry: *Elaeis oleifera* (O), native to the Americas, and *Elaeis guineensis* (G), native to Africa. This work provides to our knowledge, the first association mapping study in an interspecific OxG oil palm population, which shows tolerance to pests and diseases, high oil quality, and acceptable fruit bunch production.

**Results:**

Using genotyping-by-sequencing (GBS), we identified a total of 3776 single nucleotide polymorphisms (SNPs) that were used to perform a genome-wide association analysis (GWAS) in 378 OxG hybrid population for 10 agronomic traits. Twelve genomic regions (SNPs) were located near candidate genes implicated in multiple functional categories, such as tissue growth, cellular trafficking, and physiological processes.

**Conclusions:**

We provide new insights on genomic regions that mapped on candidate genes involved in plant architecture and yield. These potential candidate genes need to be confirmed for future targeted functional analyses. Associated markers to the traits of interest may be valuable resources for the development of marker-assisted selection in oil palm breeding.

## Background

The oil palm is an important crop that has a higher quality oil and a greater yield potential compared to other oil-producing crops [1]. Colombia is the fourth-largest oil palm producer worldwide with 1.8 million tons produced for the year 2018 and a yield of 3.8 tons/ha, placing the country above the average global yield [2]. Within the Arecaceae family, the African oil palm (*Elaeis guineensis*), native to West Africa, is the primary source of most of the vegetable oil found worldwide [3]. However, another palm species known as the American oil palm (*Elaeis oleifera*), which is native to the tropics of Central and South America, is recognized for its high yield production [3]. Both palm species are perennial monocots with lifespans of approximately 25 years [4], which results in slow breeding processes. The Corporación Colombiana de Investigación Agropecuaria (Agrosavia) established a breeding program focused on developing OxG interspecific hybrids (*E. oleifera* x *E. guineensis*). The OxG is characterized by having slow trunk growth [5], tolerance to bud rot [6–9], and red ring diseases [10] in comparison to the parent species. Additionally, these OxG populations inherited the parthenocarpic fruit development of *E. oleifera*, which allows the production of seedless fruits [11].

Saturated genetic linkage maps are essential for the identification of genomic regions associated with major genes and with quantitative trait loci (QTLs) that control agronomic traits. Over the last 20 years, multiple genetic maps of the oil palm have been constructed. The first such map was generated using restriction fragment length polymorphisms (RFLPs) and amplified fragment length polymorphisms (AFLPs) [12, 13]. Dense genetic maps were subsequently constructed using simple sequence repeats (SSRs) and single nucleotide polymorphism (SNP) markers, which have also been used for QTL identification. Using this approach, Jeennor and Volkaert [14] identified a QTL associated with bunch weight using a mapping population of 69 accessions and generated a genetic map with 89 SSRs and 101 SNPs. Further, Billotte et al. [15] used a multi-parent linkage map elaborated with 251 SSRs and reported QTLs associated with bunch traits. Similar approaches have enabled the identification of 164 QTLs associated with 21 oil yield components using SSR, AFLP, and RFLP markers [16].

In recent years, advances in next-generation sequencing technology have lowered DNA sequencing costs and thousands of SNPs have now been obtained [17, 18]. In particular, genotyping-by-sequencing (GBS) is a rapid, low-cost, and robust approach for screening breeding populations using SNPs [19]. Pootakham et al. [20] constructed an oil palm map using an F_2_ population and 1085 SNPs derived from GBS and were able to identify QTLs for height and fruit bunch weight. Similarly, a genome-wide association analysis (GWAS), using a larger number of SNPs (4031) derived from GBS across a diverse panel of *E. guineensis,* allowed the identification of novel QTLs associated with the increase in trunk height [21].

GWAS has been proposed as a much more robust approach compared to QTL linkage mapping [22]. The use of a wide range of genetic backgrounds in GWAS analyses increases the probability of detecting QTL regions associated with traits of interest, compared to the limited genetic variation of a bi-parental mapping population [23]. However, the limitations of GWAS, such as the effect of population structure, can lead to spurious associations between a candidate marker and a specific phenotypic trait [24]. To eliminate such association, the mixed linear model incorporates structure data (Q) and relative kinship effects (K), resulting in the reduction of false-positive associations [25].

Given palm oil’s use in numerous prepared foods and industrial and medical applications, the economic importance of this crop has experienced rapid growth and palm oil is now the second most traded vegetable oil world-wide after soybean [26, 27]. The demand for this crop is increasing due to a shift away from trans-fats to healthier alternatives [28], and because its residues can be processed to produce biofuel [27]. For these reasons, the identification of specific genomic regions whose genes are involved in morphological traits, such as height and foliar area, and the relationship between these traits and productivity, is becoming increasingly important for this crop.

Although previous studies have identified QTLs controlling morphological and yield-related traits in oil palm, these QTLs were detected using intraspecific populations. Our study is the first report in which molecular markers have been mapped through association analysis in an interspecific OxG population. Our study aims were: (i) genotype an OxG oil palm mapping population; and (ii) perform GWAS to identify loci or candidate genes involved in morphological and yield-related traits for future use in breeding programs.

## Results

### Analysis of phenotypic data

**Fig. 1:**
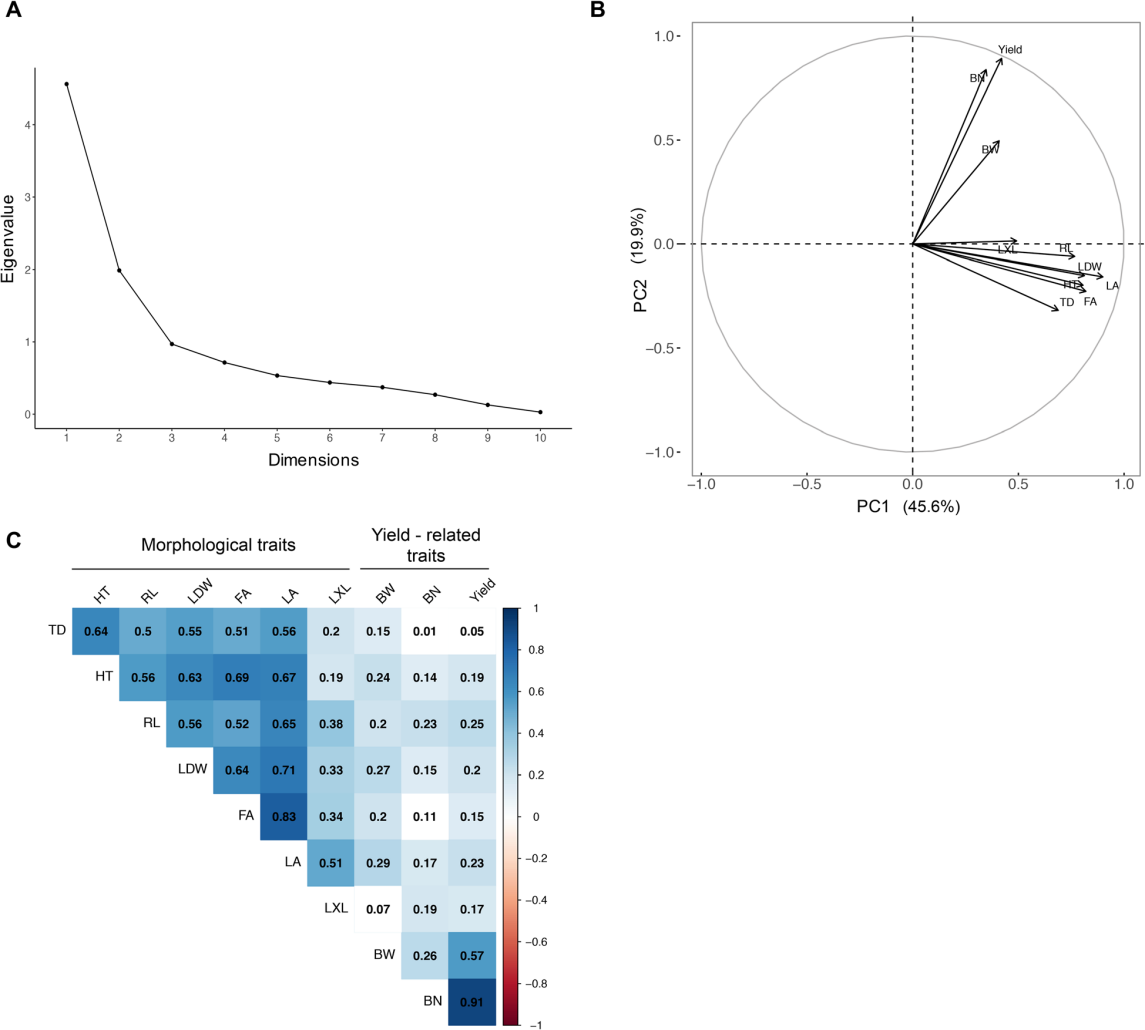
**a** Scree plot calculated across 10 traits for a population of 378 *E. oleifera x E. guineensis* (OxG) individuals; **b** Principal components loading plot for the population of 378 OxG for PC1 & PC2 calculated across 10 traits; **c** Correlation among 10 traits of the 378 OxG. TD = Trunk Diameter, HT = Trunk Height, RL = Rachis Length, LDW = Leaf Dry Weight, FA = Foliar Area, LA = Leaf Area, LXL = Leaflet per Leaf, BW = Bunch Weight, BN = Bunch number, and Yield = Yield per Palm. Color boxes indicate significant correlations (*p* ≤ 0.01), and white boxes indicate coefficients with *p* ≥ 0.01

Means, standard deviations, and range values of the phenotypic data for the population of 378 OxG hybrids are shown in Table 1. The first principal component (PC1) explained 45.6% of the total phenotypic variation, where morphological-related traits, such as leaf area (LA), foliar area (FA), leaf dry weight (LDW), and trunk height (HT) contributed extensively to this component. Meanwhile, the second principal component (PC2) explained 19.9% of the variance, associated mainly with yield-related traits (Fig. 1a-b). Positive correlations were observed between most of the morphological traits (*r* = 0.1 to 0.8), while lower correlation values were found between yield and most of the morphological traits (*r* ≤ 0.3) (Fig. 1b). Notably, HT was correlated with FA, LA, LDW and trunk diameter (TD) (*r* ≥ 0.6), whereas yield was highly correlated with bunch number (BN) (*r* = 0.91); furthermore, it also showed a weaker correlation with bunch weight (BW) (*r* = 0.57).

**Table 1:** Mean values, standard deviations (SD) and minimum and maximum values of the phenotypic traits used in this study

Category	Trait	Abbreviation	Unit	Mean	SD	Minimum value	Maximum value
Morphological	Trunk Diameter	TD	cm	88.5	6.0	62.4	102.0
	Trunk Height	HT	cm	250.3	29.5	133.3	327.0
	Rachis Length	RL	cm	421.5	35.3	275.5	530.0
	Leaf Dry Weight	LDW	kg	2.2	0.3	1.3	3.7
	Foliar Area	FA	m^2^	385.0	78.2	141.3	617.1
	Leaf Area	LA	m^2^	8.6	1.3	4.7	12.7
	Leaflet per Leaf	LXL	unit	234.8	14.8	184.0	294.0
Yield	Bunch Weight	BW	kg	6.1	1.8	1.0	19.5
	Bunch Number	BN	unit	8.8	5.0	1.0	27.0
	Yield per Palm	Yield	kg	56.5	39.1	1.8	233.0

**Fig. 2:**
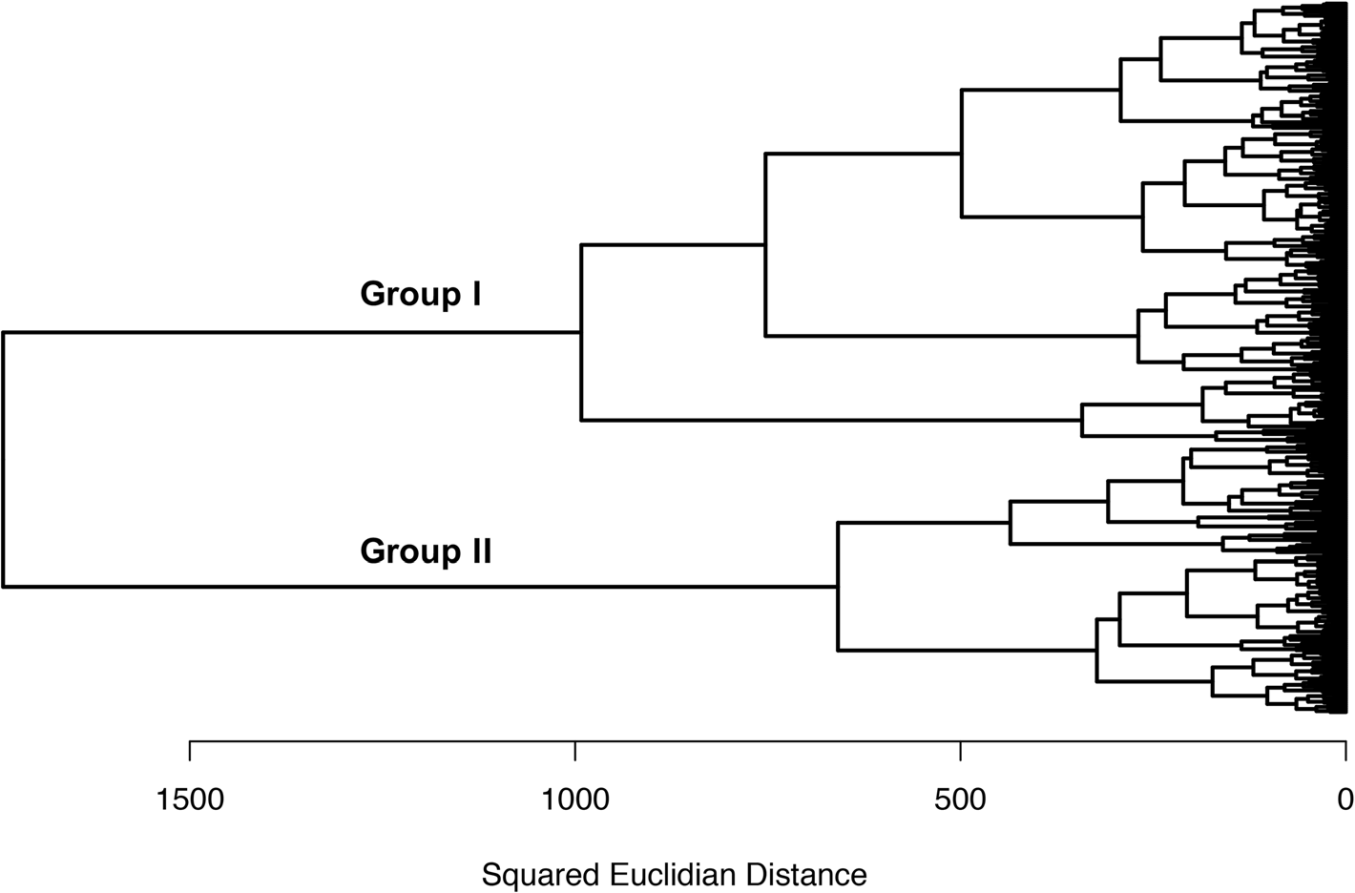
Hierarchical cluster analysis of the OxG population calculated across 10 phenotypic traits. Three hundred seventy-eight individuals were clustered using Ward’s method and the squared Euclidean distance

A hierarchical cluster analysis was performed to evaluate the phenotypic similarity among the 378 OxG hybrids (Fig. 2; Additional file 1: Table S1). We found phenotypic differences between the two clusters to agree with the variability of the morphological-related traits. Overall, Group II showed the highest mean values for all the morphological-related traits (Additional file 3: Figure S1), e.g., OxG individuals from Group II were significantly taller (HT = 269 ± 21 cm) compared to OxG from Group I (HT = 238 ± 28 cm) (*p* ≤ 0.0001). However, yield-related traits had no significant differences between groups.

### SNP calling

A total of 1,058,182,456 raw Illumina sequencing reads from seven Illumina HiSeq lanes were generated for 471 palms (62 *E. oleifera* (O)*,* 31 *E. guineensis* (G)*,* and 378 (OxG)). The genotyping of the collection detected 131,825 SNPs covering 16 oil palm chromosomes. After filtering, 3776 SNPs with an average of 236 SNPs per chromosome were retained (Additional file 2: Table S2).

### Cluster and association analyses

**Fig. 3:**
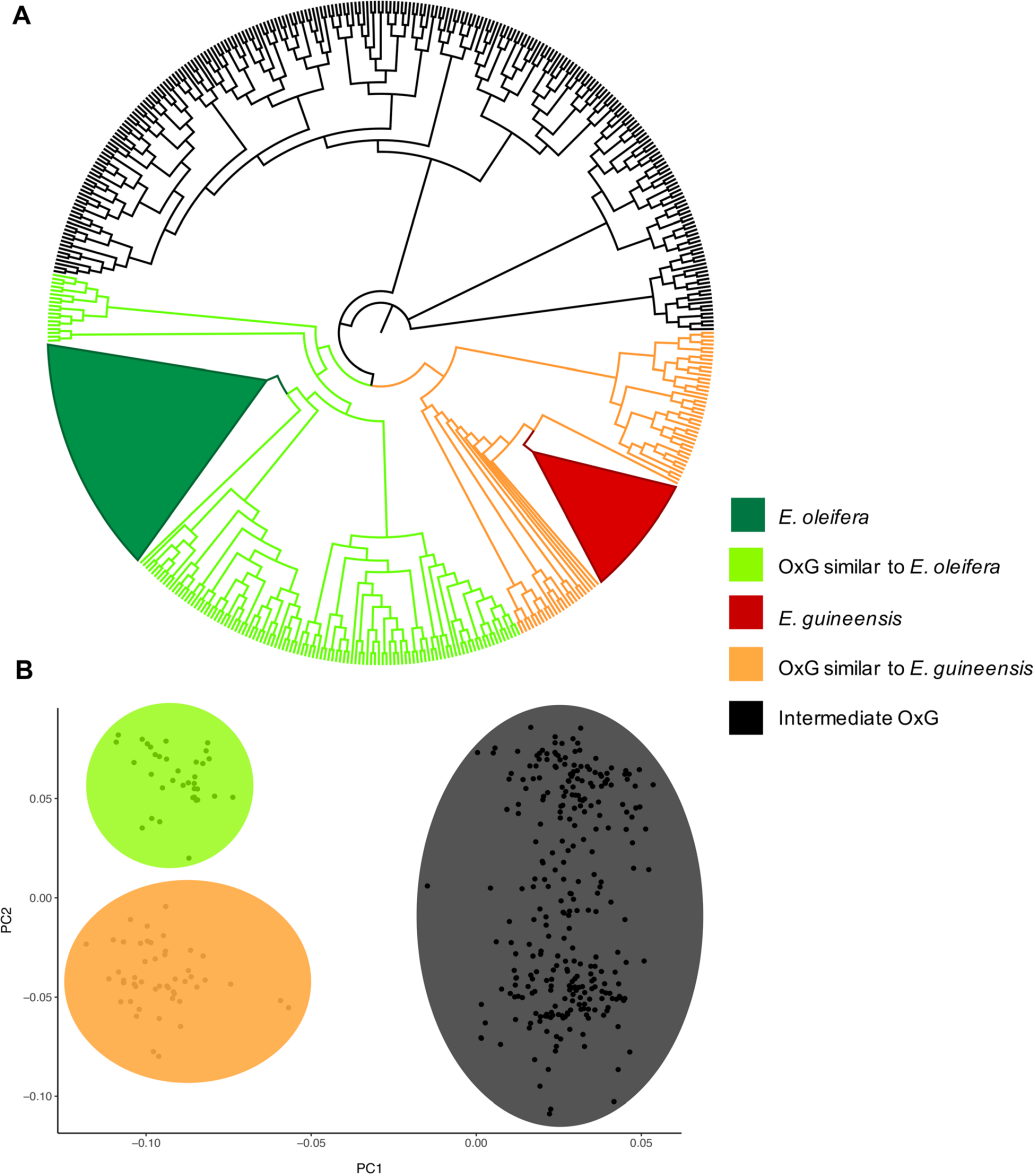
**a** Neighbor-Joining tree of 471 diverse oil palms (62 *E. oleifera* (O)*,* 31 *E. guineensis* (G)*,* and 378 (OxG)) based on Nei’s genetic distance; **b** Principal component analysis (PCA) of 378 individuals of the OxG population separated into two groups. Both analyses were based on 3776 SNPs

The neighbor-joining (NJ) analysis of the entire population (471 palms) (Fig. 3a) showed two main groups containing *E. oleifera* and *E. guineensis,* as well as three groups within the OxG population, as follows: One group was more similar to *E. guineensis*, another was more similar to *E. oleifera*, and the largest group showed an intermediate similarity to both parental species. The three groups in the OxG population represented the classic distribution of crosses between two highly heterozygous diploids (Aa x Aa) with a genotypic segregation ratio of 1:2:1.

The PCA analysis of the OxG population (378 hybrids) showed that the first three components comprised approximately 15.47% of the total variation and allowed the population to be categorized into three groups, thereby supporting the results observed in the NJ tree in accordance with the segregating nature of our population (Fig. 3b).

**Fig. 4:**
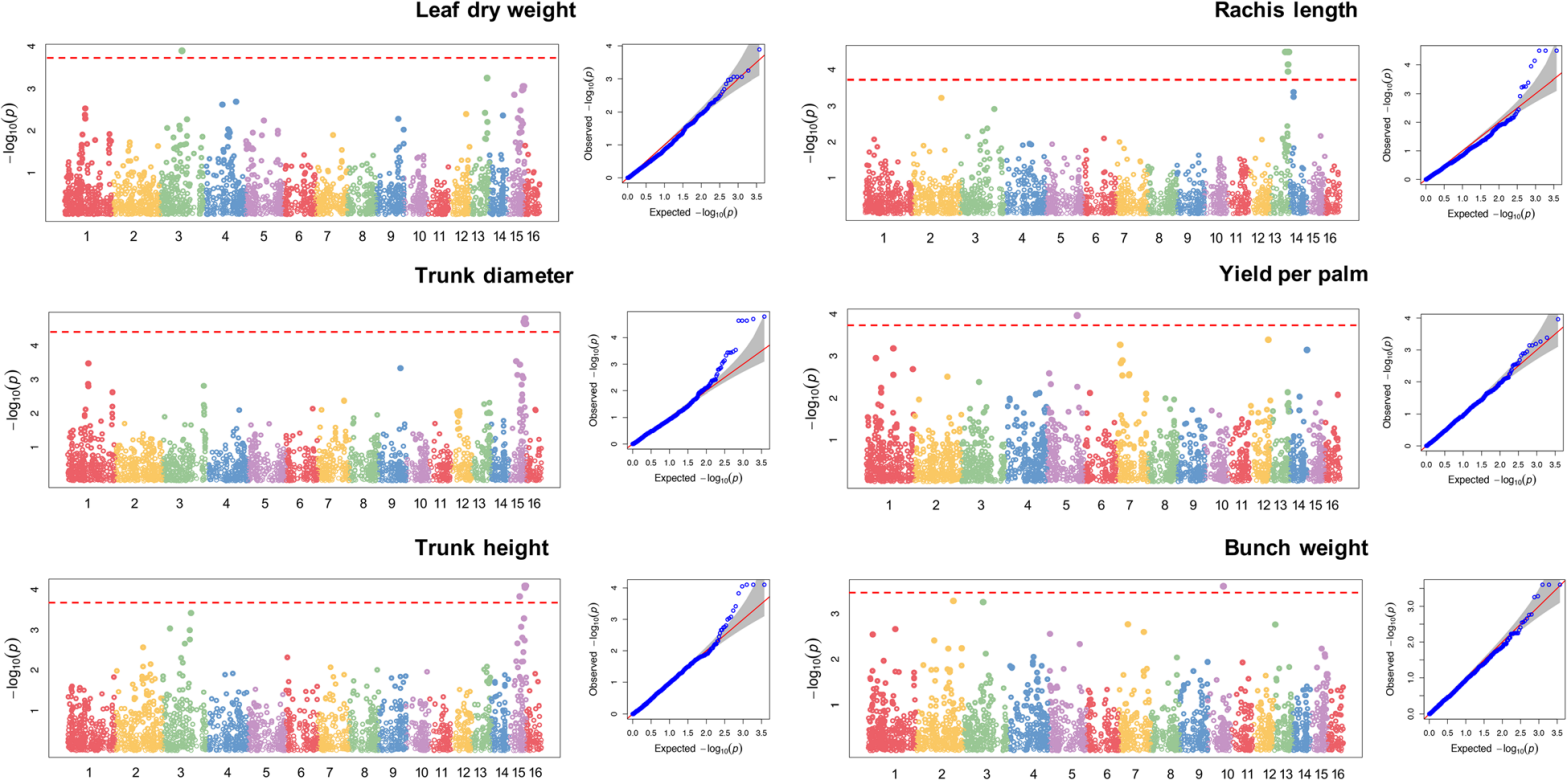
Manhattan and Q-Q plots of the 378 OxG population, indicating genomic regions associated with leaf dry weight (LDW), trunk diameter (TD), trunk height (HT), rachis length (RL), yield per palm, and bunch weight (BW). The red horizontal line indicates the significant association threshold

We performed the association analysis on the 378 OxG hybrids and 3776 SNPs for seven morphological traits and three yield-related traits (Table 1). Twelve SNPs were most significantly associated with the traits measured, based on *p*-values across different genomic regions of the oil palm genome before the false discovery rate (FDR) correction (Table 2). Common SNPs for rachis length (RL) and leaflet per leaf (LXL) were observed, as well as for HT and LA, and between yield and BN, following the results from the phenotypic correlations. The Q-Q plots (Fig. 4) significantly supported the evidence for SNP associations with the traits (*p* ≤ 0.005) and suggested that population stratification in the GWAS model was adequately controlled.

**Table 2:** Significant marker-trait associations for 378 individuals of the OxG population for morphological and yield-related traits using a mixed linear model approach

Category	Trait	SNP	Chromosome	Position (kb)	*p*-value	MAF	R^2^	FDR adjusted *p*-values	Nearby gene	SNP position relative to the candidate gene*	Candidate gene annotation
Morphological	LDW	S3_30,467,222	3	30,467,222	1.E-04	0.106	0.107	0.050	p5.00_sc00100_p0017	0	Mechanosensitive ion channel protein 10-like (MSL10)
	TD	S15_21,239,833	15	21,239,833	1.98E-05	0.493	0.097	0.010	p5.00_sc00036_p0097	+ 21.8 kb	Nucleic acid binding
	HT TD FA LA	S15_22,347,191	15	22,347,191	7.94E-05	0.496	0.098	0.084	p5.00_sc00036_p0145	+ 0.3 kb	Paired amphipathic helix protein (PAH)
		S15_22,553,489	15	22,553,489	7.94E-05	0.496	0.098	0.084	p5.00_sc00036_p0152	−0.6 kb	Serine threonine-protein kinase (STYK)
		S15_22,553,493	15	22,553,493	8.90E-05	0.495	0.098	0.084			
		S15_23,645,020	15	23,645,020	7.94E-05	0.496	0.098	0.084	p5.00_sc00036_p0217	0	Class E vacuolar protein-sorting machinery protein (VPS)
	RL LXL	S13_20,856,724	13	20,856,724	3.22E-05	0.499	0.074	0.041	p5.00_sc00035_p0180	−12.0 kb	Guanine nucleotide-binding protein subunit gamma (AGC3)
		S13_23,674,227	13	23,674,227	3.22E-05	0.499	0.074	0.041	p5.00_sc00035_p0078	0	Extracellular ribonuclease (RNAse)
		S13_25,522,088	13	25,522,088	3.22E-05	0.499	0.074	0.041	p5.00_sc00128_p0001	0	lmbr1 domain-containing protein (IMBR1)
		S13_24,474,516	13	24,474,516	7.14E-05	0.497	0.070	0.067	p5.00_sc00035_p0043	0	Probable ran guanine nucleotide release factor-like (RANGRF)
Production	Yield BN	S5_41,396,842	5	41,396,842	1.E-04	0.132	0.059	0.410	p5.00_sc00003_p0367	+ 29 kb	Cation h(+) antiporter
	BW	S10_21,597,426	10	21,597,426	3.E-04	0.499	0.054	0.316	p5.00_sc00036_p0097	−8.0 kb	Zinc finger protein 8-like (ZFP)

^*^ SNP position relative to the closest candidate gene: upstream and downstream SNPs of candidate genes are specified with “–” and “+,” respectively. 0 indicates that SNPs are located within the candidate gene

The availability of the oil palm genome sequence [29] enabled the association of specific QTLs with genomic regions on the physical map and the exploration of potential candidate genes and their possible functions. On chromosomes 3, 13, and 15, we identified 10 significant SNPs located on genomic regions harboring genes associated with the morphological-related traits before the FDR correction (Fig. 4 and Table 2). For yield-related traits, we observed two SNPs into two candidate genes on chromosomes 5 and 10, which were non-significant after carrying out the FDR correction (Fig. 4, Table 2). We evaluated whether the SNPs found in association with traits were in chromosomes with a larger number of markers to assess whether our results could have arisen from biases in the genotyping. The associated SNPs found in this study (chromosomes 3, 5, 10, 13, and 15) were not located in the chromosomes with higher numbers of SNPs as identified by the GBS approach (Additional file 2: Table S2).

The pair-wise linkage disequilibrium (LD) between the SNPs of the chromosomes that were presented in the genomic regions associated with the evaluated traits is illustrated in Additional file 4: Figure S2. The LD blocks were small for all chromosomes shown, which was expected, considering the out-crossing nature of the species.

## Discussion

Improving oil quality and increasing yield per hectare in oil palm are major concerns in the oil processing industry. The Corporación Colombiana de Investigación Agropecuaria (Agrosavia), a non-profit government research institution, is committed to delivering solutions to farmers, incorporating cultivars developed from breeding programs that include the oil palm. Its strategy has focused on developing interspecific OxG that present heterosis in traits such as resistance to diseases, fruit number, fruit weight, leaf length, and trunk diameter [30]. To our knowledge, this study is the first GWAS analysis of an OxG population.

### Phenotypic data

Correlation analysis results for yield-related traits indicated that BN could have the potential to be a better selection criterion for production compared to BW in the OxG population. In our study, no significant correlations between yield and leaf-related traits (FA, LA, LDW, LXL, RL) were found; however, a previous study in *E. oleifera* and with OxG hybrids found that BN can be higher than the number of leaves, but only at the time when oil palms are producing multiple inflorescences [31]. Increases in BN and BW are also expected to correlate with increased mesocarp and kernel oil yields, as shown in other oil palm germplasm studies [32]. Future studies directed to improve the oil yields should be conducted considering the importance of this aspect of oil palm breeding.

### Association analysis

In the current study, we generated sequencing data using GBS, a technology developed for crop plants [19]. GBS relies on restriction enzymes to generate a reduced representation of locations spread throughout the genome to decrease its complexity and rapidly genotype samples using interspaced SNP markers [33], that could be linked to candidate genes responsible for important traits. For this reason, GBS has gained popularity in crop research and plant breeding due to its high throughput and low-cost genotyping, being suitable for population studies, germplasm characterization, genetic improvement, and trait mapping in a variety of diverse organisms [34].

With the association mapping, 12 genomic regions (SNPs) related to 10 morphological and yield-related traits were identified (Table 2). However, only five regions associated with LDW, TD, RL, and LXL remained significant (*p* ≤ 0.05) after the FDR correction was performed. Importantly, the SNPs found to have a statistically significant association with the trait are not necessarily the causal DNA variant, that is, a variant that has a direct effect. The association only signifies that the SNP locus harbors a causal variant in LD with the SNP identified by the GWAS.

The small LD blocks in the heat map analysis could suggest that the causal regions are located near to the most significant SNPs. Thus, the identified SNP in this study serves as a signpost defining an interval in the genome for which one must do follow-up studies to determine the causal variant(s).

Therefore, we describe the five most significant regions and the genes located within those regions that might be potential candidate genes involved in the expression of the phenotypic traits evaluated in this study. For morphological traits, a significant association was found for LDW on chromosome 3, explaining 10% of the phenotypic variation. The most significant SNP in this region was located in a mechanosensitive (MS) ion channel protein 10-like (*MSL10*) gene. It has been proposed that the MS ion channels in plants play a wide array of roles, from facilitating the perception of touch and of gravity to regulating the osmotic homeostasis of intracellular organelles [35]. In addition, mechanoperception genes are essential for the growth and development of normal cells and tissue as well as for the proper responses to an array of biotic and abiotic stresses [36]. A second significant region was identified associated with TD on chromosome 15 that contains a gene involved in nucleic acid binding that has a C2H2-type zinc finger domain. It has been proposed that the C2H2-ZF gene family is involved in the formation of wood and in shoot and cambium development in species such as poplar, and that it also plays a role in stress and phytohormone responses [37].

For RL and LXL traits, QTLs have been reported on chromosomes 2, 4, 10, and 16 [32]. In our study, three SNPs were associated with three different candidate genes for RL on chromosome 13. The SNP S13_20,856,724 is the closest to the *AGC3* gene and encodes different G proteins. These have been reported to be involved in a wide range of developmental and physiological processes, and therefore have a potential for facilitating yield improvement in crops such as rice [38]. The second significant association was found with the SNP S13_23,674,227, which is located in an extracellular ribonuclease gene (*RNase* gene). The *RNase* genes in plants have been studied for years and play an essential role in plant defense [39] and development due to their ability to modify RNA levels and thereby influence protein synthesis [40]. Finally, the SNP S13_25,522,088 was also significantly associated with RL and LXL, but further studies are necessary to determine its role, if any, in regulating these traits.

Seven SNPs were no longer significant after the FDR correction, possibly due to the reduced sample size used. QTL and association studies are limited by the relatively small mapping population sizes, resulting in low statistical power and thus rendering small or even medium-effect QTLs that are statistically non-significant and difficult to detect. Such statistically underpowered populations may also suffer from severe inflation of effect size estimates (the so-called Beavis effect) [41]. Hence, increasing the population size and marker density is required to enable estimations that are unbiased by the Beavis effect and achieve higher statistical power [41–43]; nonetheless, for perennial populations (long generation time) with limited offspring numbers, the size increase would require a considerable investment.

For the oil palm, the harvesting of fruit bunches after the palm has reached a certain age is an arduous task due to the height of the trunk. For this reason, genotypes with reduced HT and TD are preferred among oil palm farmers. Likewise, a larger foliar area (dependent on RL and LDW) is related to greater photosynthetic production, which could be involved in higher productivity. Nevertheless, most importantly, increasing the number and weight of fruits means a higher productivity per palm and therefore a higher income for farmers. For this reason, leveraging QTLs or genes related to these traits (such the ones we identify in this study) could contribute to the development of plant breeding strategies, such as marker-assisted selection that help with the selection of promising accessions in earlier stages (i.e., greenhouse conditions) and therefore reduce the breeding cycle. There is need for further work that focuses on the biological functions of the set of potential candidate genes found in our research since the correlations we have identified in our association study cannot, as yet, be dubbed as causations.

## Conclusions

Our study is the first to report five significant genomic regions associated with morphological and yield-related traits based upon GWAS on an interspecific OxG oil palm population. Genes whose functional annotations are potentially related to the corresponding traits are located within these regions and, therefore, these might represent candidate genes for the QTLs. Our results will provide the groundwork for the development of marker-assisted breeding in the oil palm and will serve as a strong base for future functional studies to determine the drivers of high yield production.

## Methods

### Plant material

A total of 471 diverse oil palms (62 *E. oleifera* (O) accessions*,* 31 *E. guineensis* (G) accessions*,* and 378 OxG hybrids) from the El Mira and La Libertad research centers of the Corporación Colombiana de Investigación Agropecuaria (Agrosavia) [44], were included in this research. The OxG population was obtained through eight different crossings (eight different *E. oleifera* accessions as female progenitors were crossed with one *E. guineensis* accession as the male progenitor); however, the parents of these crossings are currently dead. Details of the crosses and the origins of individuals are given in Additional file 1: Table S1. The plant material belongs to the National Germplasm Collection of Colombia maintained by Agrosavia. All samples were collected following national regulations.

### Phenotyping

Phenotypic data were collected for the subset of 378 OxG hybrids, that were planted in a quincunx or triangular system with 10 m between the plants at El Mira research center of Agrosavia in Tumaco, Colombia. Plants were randomly distributed using a completely randomized block design with four blocks.

A total of 10 traits (Table 1) distributed between two categories (morphological and yield-related), were evaluated as follows: i) Morphological category (seven traits): Trunk Diameter (TD, trunk circumference at the midsection), Trunk Height (HT, distance between the lowest green leaves and the fruit), Rachis Length (RL, measured on fully expanded leaves), Leaf Dry Weight (LDW, mean dry weight per leaf multiplied by the number of leaves produced), Foliar Area (FA, mean area per leaf multiplied by the number of leaves per palm), Leaf Area (LA, mean area per leaf), and Leaflet per Leaf (LXL, length of the largest leaflet). ii) Yield-related category (three traits): Bunch Weight (BW, the weight of fruits during harvest), Bunch number (BN, the number of fruits per palm during harvest), and Yield per Palm (Yield, kg of fruits per palm per year). Each trait was measured according to the methodology presented by Corley et al. [45] and Breure [46].

### Statistical analysis of phenotypic data

The correlations among traits were calculated using Pearson’s correlation coefficient (*r*) with *p* ≤ 0.05. To assess the relationships between the studied traits, a principal component analysis (PCA) was carried out. Finally, a hierarchical cluster analysis using Ward’s method was carried out to analyze the relationships between hybrids. Differences between clusters by trait were established using a *t*-test with *p* ≤ 0.0001*.* All statistical analyses were performed using the R v3.42 software [47].

### Genotyping

Genomic DNA of 471 palms was extracted from leaf tissue using the DNeasy Plant Mini Kit (QIAGEN, Germany). The DNA quality was estimated using the *Hind*III enzyme and visualized by electrophoresis on 2% agarose gels. The GBS libraries were constructed with the methylation-sensitive restriction enzyme *PstI* (CTGCAG). Sequencing was performed with 100-bp single-end reads using the Illumina HiSeq 2000 platform (Illumina Inc., United States) at the Institute of Genomic Diversity (Cornell University, Ithaca, NY, United States).

### SNP discovery and data processing

Illumina reads were demultiplexed using the standard pipeline from Tassel v4.5.9 software [48]. Then, reads were mapped to the oil palm reference genome of *E. guineensis* [49] using Bowtie2 [50] employing the *very-sensitive* option. SNP calling was performed using the following parameters: minor allele frequency (MAF) < 5%, minimum locus coverage (mnLCov) of 0.9, minimum site coverage (mnScov) of 0.7 and minimum taxon coverage (mnTCov) of 0.5. Finally, SNPs were filtered using the VCFtools v0.1.13 software [51] to remove 95% of missing data and to retain biallelic SNPs.

### Cluster and marker-trait association analyses

The clustering analysis for all 471 oil palms was performed by a neighbor-joining algorithm using Tassel v4.3.5 [48] and was visualized with Figtree v1.4.0 [52]. The population structure for the 378 OxG hybrids was evaluated through a PCA using the SNPrelate [53] procedure in the R package. Associations between molecular markers and phenotypic data were computed using the mixed linear model in the software GAPIT (Genome Association and Prediction Integrated Tool) [54]. To avoid any possible bias caused by population structure, we included the first five principal components of the PCA and a relatedness (kinship) matrix from GAPIT in the mixed linear model. Quantile-quantile (Q-Q) plots using the observed −log_10_ *p*-values and the expected −log_10_ *p*-values were generated to study the appropriateness of the GWAS model. A false discovery rate (FDR) [55] was used to correct for spurious associations.

The heat map of the linkage disequilibrium (LD) was generated with a custom script by plotting pairwise R^2^ values against the physical distance (base pairs) between markers on the same chromosome.

### Potential candidate gene identification

The physical positions of the SNP markers were obtained from the Genomsawit website of the International Malaysian Oil Palm Genome Programme (http://gbrowse.mpob.gov.my/fgb2/gbrowse/Eg5_1/). Gene annotations under the candidate gene regions were established using published genome information for *E. guineensis* [49]. The flanking sequences of SNPs to assign the putative biological functions of significant SNP markers associated with the traits were queried against databases, such as HMMER (https://www.ebi.ac.uk/Tools/hmmer/) and NCBI (http://www.ncbi.nlm.nih.gov/), and those of the European Molecular Biology Laboratory (http://www.ebi.ac.uk/) and the European Nucleotide Archive (http://www.ebi.ac.uk/ena).

## Supplementary information

**Additional file 1: Table S1.** List of the OxG and parentals oil palms used in this study.

**Additional file 2: Table S2.** SNPs identified per chromosome using the reference genome of *E. guineensis*.

**Additional file 3: Figure S1.** Box plots of the two cluster groups for all morphological and yield-related traits. * = significant at *p* ≤ 0.0001, ns = non-significant.

**Additional file 4: Figure S2.** Linkage disequilibrium (LD) heat map for each chromosome with significant associated SNPs in an OxG population.

## Data Availability

The datasets used and analyzed during the current study are available from the corresponding author upon reasonable request.

## Abbreviations

AFLP: Amplified Fragment Length Polymorphism
BN: Bunch Number
BW: Bunch Weight
FA: Foliar Area
FDR: False-Discovery-Rate
GBS: Genotyping-By-Sequencing
GWAS: Genome-Wide Association Studies
HT: Trunk Height
LA: Leaf Area
LD: Linkage Disequilibrium
LDW: Leaf Dry Weight
LXL: Leaflet Per Leaf
PCA: Principal Component Analysis
QTL: Quantitative Trait Loci
RFLP: Restriction Fragment Length Polymorphism
RL: Rachis Length
SD: Standard Deviation
SNP: Single Nucleotide Polymorphism
SSR: Simple Sequence Repeat
TD: Trunk Diameter
