# Supplementary figures and images for: Genome-wide association study (GWAS) for morphological and yield-related traits in an oil palm hybrid (Elaeis oleifera x Elaeis guineensis) population
Source: BMC Plant Biol. 2019 Dec 3;19:533. doi: 10.1186/s12870-019-2153-8 (PMC6889324; doi:10.1186/s12870-019-2153-8)

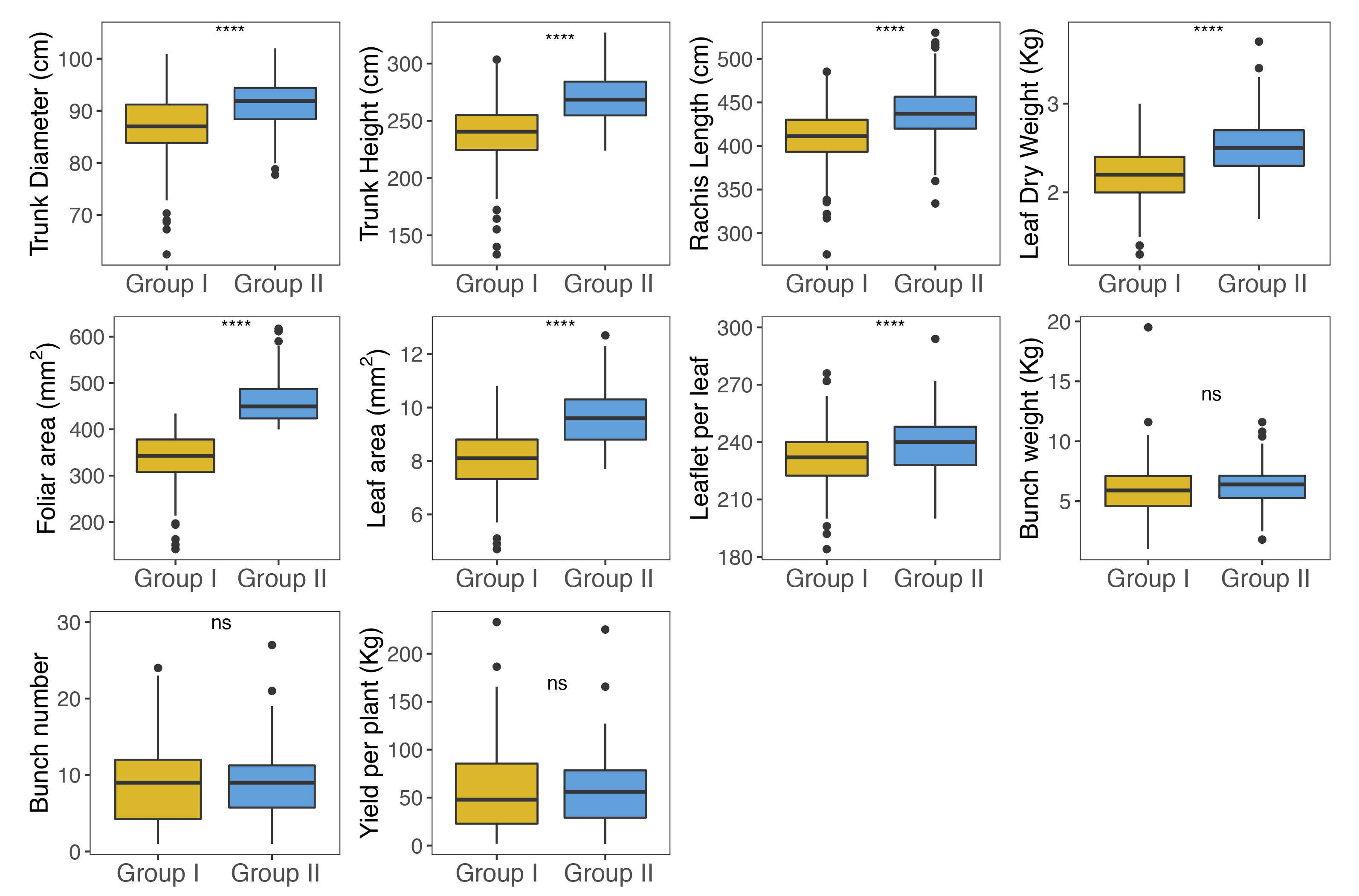

Supplement: Supplementary file 3 — Additional file 3: Figure S1. Box plots of the two cluster groups for all morphological and yield-related traits. * = significant at p ≤ 0.0001, ns = non-significant. [file 12870_2019_2153_MOESM3_ESM.tif]

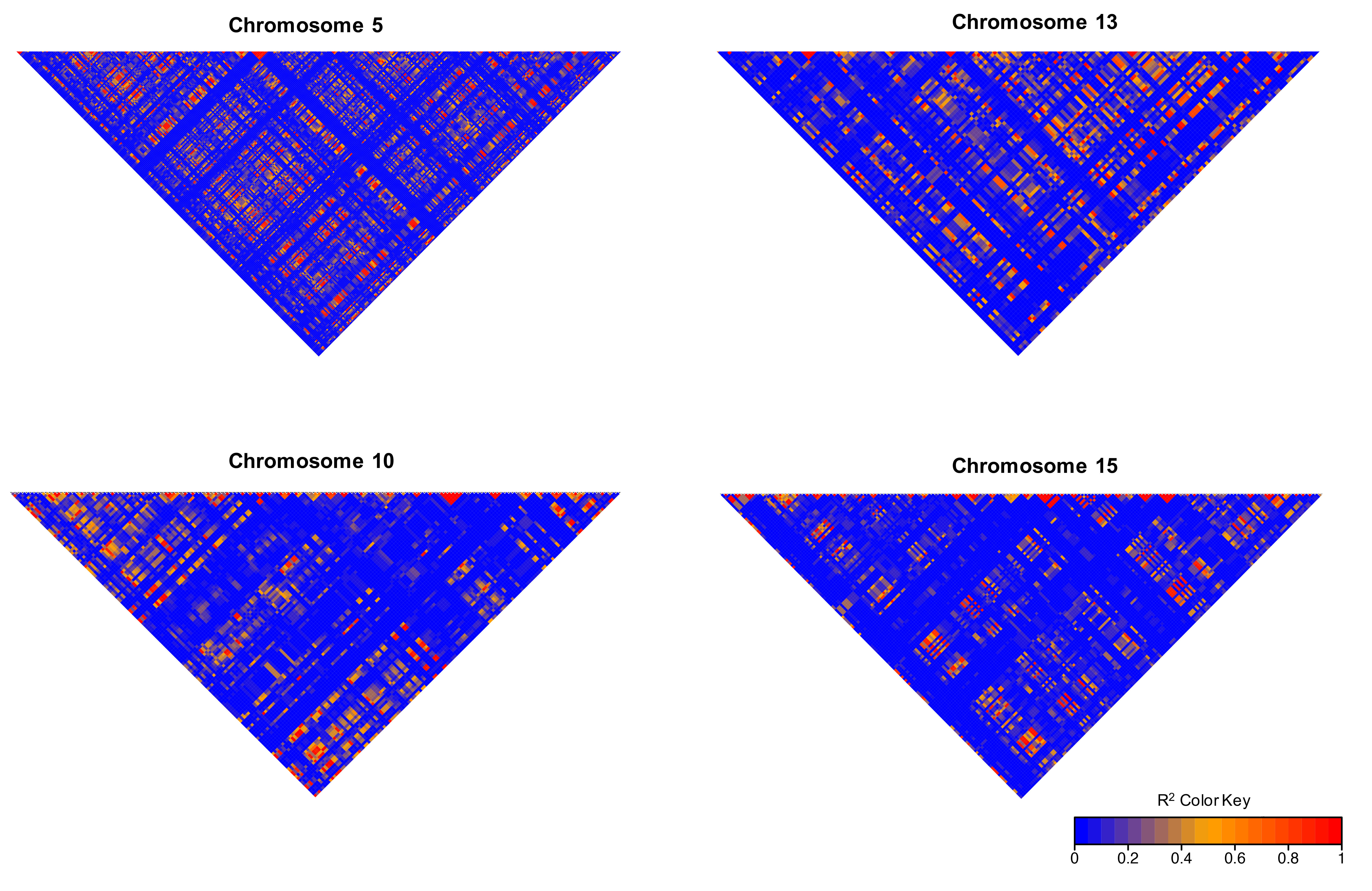

Supplement: Supplementary file 4 — Additional file 4: Figure S2. Linkage disequilibrium (LD) heat map for each chromosome with significant associated SNPs in an OxG population. [file 12870_2019_2153_MOESM4_ESM.tif]
